# Distinct *C9orf72*-Associated Dipeptide Repeat Structures Correlate with Neuronal Toxicity

**DOI:** 10.1371/journal.pone.0165084

**Published:** 2016-10-24

**Authors:** Brittany N. Flores, Mark E. Dulchavsky, Amy Krans, Michael R. Sawaya, Henry L. Paulson, Peter K. Todd, Sami J. Barmada, Magdalena I. Ivanova

**Affiliations:** 1 Cellular and Molecular Biology Graduate Program, University of Michigan, Ann Arbor, Michigan, United States of America; 2 Department of Neurology, University of Michigan, Ann Arbor, Michigan, United States of America; 3 UCLA-DOE Institute for Genomics and Proteomics, University of California, Los Angeles, California, United States of America; 4 Neuroscience Graduate Program, University of Michigan, Ann Arbor, Michigan, United States of America; 5 Veterans Affairs Medical Center, Ann Arbor, Michigan, United States of America; 6 Biophysics Program, University of Michigan, Ann Arbor, Michigan, United States of America; Children's Hospital of Pittsburgh, University of Pittsburgh Medical Center, UNITED STATES

## Abstract

Hexanucleotide repeat expansions in *C9orf72* are the most common inherited cause of amyotrophic lateral sclerosis (ALS) and frontotemporal dementia (FTD). The expansions elicit toxicity in part through repeat-associated non-AUG (RAN) translation of the intronic (GGGGCC)_n_ sequence into dipeptide repeat-containing proteins (DPRs). Little is known, however, about the structural characteristics and aggregation propensities of the dipeptide units comprising DPRs. To address this question, we synthesized dipeptide units corresponding to the three sense-strand RAN translation products, analyzed their structures by circular dichroism, electron microscopy and dye binding assays, and assessed their relative toxicity when applied to primary cortical neurons. Short, glycine-arginine (GR)3 dipeptides formed spherical aggregates and selectively reduced neuronal survival compared to glycine-alanine (GA)3 and glycine-proline (GP)3 dipeptides. Doubling peptide length had little effect on the structure of GR or GP peptides, but (GA)6 peptides formed β-sheet rich aggregates that bound thioflavin T and Congo red yet lacked the typical fibrillar morphology of amyloids. Aging of (GA)6 dipeptides increased their β-sheet content and enhanced their toxicity when applied to neurons. We also observed that the relative toxicity of each tested dipeptide was proportional to peptide internalization. Our results demonstrate that different *C9orf72*-related dipeptides exhibit distinct structural properties that correlate with their relative toxicity.

## Introduction

A GGGGCC repeat expansion in the first intron of *C9orf72* is the most common known inherited cause of amyotrophic lateral sclerosis and frontotemporal dementia (C9ALS/FTD) [1, 2]. This mutation explains up to 40% of all familial cases of ALS and FTD and 7% of all sporadic cases of these conditions. Most people have less than 28 repeats but in patients with expansions, hundreds to thousands of pathologic repeats are observed [3, 4].

Three mechanisms have been proposed for neurodegeneration in association with (GGGGCC)_n_ repeats in the *C9orf72* locus: (a) inhibition of *C9orf72* transcription and expression due to enhanced methylation and/or formation of DNA/RNA hybrids [1, 5–7] (b) sequestration of essential RNA-binding proteins and other factors through the formation of insoluble RNA foci by sense (GGGGCC)_n_ and antisense (GGCCCC)_n_ repeat RNAs [1, 8–11] and (c) repeat associated non-AUG (RAN) initiated translation of (GGGGCC)_n_ and (GGCCCC)_n_ repeat RNAs and the resulting accumulation of neurotoxic RAN dipeptide repeat proteins or DPRs [9, 12, 13, 14, 15–18]. Although more than one of these mechanisms may contribute to neuronal loss and disease features, we focus here on the biochemical properties of the dipeptide units comprising DPRs as evidence increasingly supports a key role for DPRs in disease pathogenesis [8, 16, 17, 19].

Both sense and antisense DPRs result from the translation of GGGGCC expansion repeats. All five of the potential DPRs (glycine-alanine GA-, glycine-proline GP-, glycine-arginine GR-, alanine-proline AP- and proline-arginine PR-) have been reported in disease brain [8, 16], though the sense strand DPRs containing GP or GR appear to be most abundant [9, 20]. Relatively little is known about the structure and morphology of DPR aggregates. Most of what is known is based on their morphological appearance in cellular models and patient tissues, except for the GA- containing peptide which was recently reported to have amyloid properties [14]

DPRs generated via AUG-initiated translation are toxic in yeast, cultured cells and *Drosophila*, and this toxicity is largely independent of (GGGGCC)_n_ RNA repeat sequences [12, 13, 17, 19, 21–28]. While several studies suggest that GR- and PR- repeat proteins are among the most toxic DPRs, GA-containing peptides are more common in individuals carrying *C9orf72* expansions, and GA-containing peptides demonstrated selective toxicity in neuronal cell lines and primary neurons [9, 14, 17, 19–30]. In contrast, no toxicity was observed with constructs selectively expressing GP- or PA- DPRs [17, 23]. These features, together with the distinct composition and distribution of DPRs in C9ALS/FTD brains [8, 16, 20], suggest that each DPR possesses unique characteristics that can be best understood by utilizing different model systems.

Defining the structural characteristics of pathogenic proteins in neurodegenerative diseases has yielded important insights into the mechanisms of inclusion formation and disease pathogenesis [31–33]. Despite the prevalence of DPRs in C9ALS/FTD pathology and their demonstrated toxicity *in vitro*, however, the structure of DPRs and how their structure relates to downstream toxicity remain unknown. Investigations of the minimal peptide segments that are the building blocks of amyloid fibrils have deepened our understanding of neurodegenerative disease pathogenesis and the aberrant behavior of disease-associated proteins [34–37]. Structural studies of these minimal units have the potential to uncover fundamental connections between protein structure and neurotoxicity, and accelerate the design of molecules that can prevent their formation.

Here we show that each of the three sense strand-derived dipeptide units underlying DPRs (GP-, GA-, and GR-), exhibits distinct structural properties that correlate with neurotoxicity when applied externally to primary rodent neurons. Using anti-DPR antibodies and fluorescence labeling, we also show that aggregates formed by the two toxic dipeptides, GA and GR, are internalized by neurons. These data suggest that short stretches of repeating GA- and GR-dipeptides can adopt structural features with the potential to impact neuronal survival.

## Materials and Methods

### Aggregation assays

All peptides were purchased from GenScript. Prior all experiments, we dissolved (GA)3, (GP)3 and (GR)3 to 20 mM and (GA)6, (GP)6 and (GR)6 to 10mM. For transmission electron microscopy (TEM), Congo red (CR), and toxicity assays, peptides were solubilized in 25mM sodium phosphate pH 7.4, 0.1M NaCl. For circular dichroism (CD), peptides were solubilized in 25mM sodium phosphate pH 7.4. (GA)3, (GP)3, (GP)6, (GR)3 and (GR)6 were soluble and their solutions remained clear after incubation. (GA)6 was not fully soluble at the concentrations used. Freshly dissolved solutions (termed “fresh”) were analyzed immediately after dissolving and were compared with samples (termed “aged”) that were instead incubated at 37°C with agitation in an EchoTherm Orbital mixer (level 9, Torrey Pines Scientific) for 6–8 days. For toxicity, CR and CD, samples were additionally diluted as described in the following sections.

Both sonicated and unsonicated samples were analyzed by CD, CR binding and TEM. Prior sonication, the peptides were additionally diluted to 4mM ((GA)3, (GP)3 and (GR)3) and 2mM ((GA)6, (GP)6 and (GR)6). The peptide samples were sonicated using continuous mode at level 10 (output power 7–8 Watts) for 15 sec by immersing the micro-probe of 60 Sonic Dismembrator (Fisher Scientific). The probe was washed with water for 1 min between samples. Data from sonicated and unsonicated peptides were reproduced in three independent experiments, yielding similar results.

### Thioflavin T (ThT) binding assay

ThT was added to each of the peptide solutions (20 mM (GA)3, (GP)3 and (GR)3 and 10mM (GA)6, (GP)6 and (GR)6 dissolved in 25mM sodium phosphate pH 7.4, 0.1M NaCl) at a final concentration of 10μM, and 110 μl of this mixture was dispensed into each well of Falcon 96-well plate (black/clear, flat bottom, Corning, 353219). Plates were then incubated at 37°C in FLUOstar Omega (BMG Labtech Inc) and were shaken at 200rpm using meander corner well shaking mode. Fluorescence was measured with gain set at 90%, an excitation wavelength of 440 nm and emission wavelength of 490 nm. Four separate replicates were measured for each sample.

### Transmission electron microscopy (TEM)

Negatively stained specimens for TEM were prepared by applying 5 μl of sample (20mM (GA)3, (GP)3, and (GR)3 and 10mM (GA)6, (GP)6, and (GR)6) to hydrophilic 400 mesh carbon-coated Formvar support films mounted on copper grids (Ted Pella, Inc., 01702-F). The samples were allowed to adhere for 4 min, rinsed twice with distilled water, and stained for 60–90 sec with 5 μl of 1% uranyl acetate (Ted Pella, Inc.). All samples were imaged at an accelerating voltage of 60 kV in a CM-100 electron microscope (Philips, Inc.). At least four different grids from four independent experiments were examined for fresh and aged samples.

### X-ray powder diffraction of (GA)6

Aged (GA)6 samples were centrifuged at 20,000 x g for 3 min. The pellet was washed twice with water, resuspended in 5 μl water, and placed between two fire-polished silanized glass capillaries (1 mm apart) and dried at room temperature. Diffraction patterns were collected using a Rigaku FRD rotating anode X-ray generator and an RAXIS-4++ imaging plate detector. Samples were oscillated 1° over a 5 min exposure.

### Circular dichroism (CD)

CD experiments were performed with Jasco J-1500 spectrometer (JASCO Analytical Instruments). The samples were additionally diluted with 25mM sodium phosphate pH 7.5 to 0.6 mg/ml ((GA)3, (GR)3, (GA)6 and (GR)6) or 0.225 mg/ml ((GP)3 and (GP)6) before measuring their spectra from 260 nm to 195 nm. Spectra were collected using scanning speed of 200nm/min, data interval of 0.5nm data, response time of 1 sec and bandwidth of 1 nm. Four spectra were measured for each sample, and data with a heat tension less than 500 were averaged. Secondary structure was predicted using programs described in [38].

### Congo red (CR) birefringence

For birefringence analysis, aged (GA)6 was pelleted by centrifugation at 20,000 x *g* for 1 min. 120μM CR (Sigma, C-6767) was added to the precipitate in 25mM sodium phosphate pH 7.4, 0.1M NaCl for 30 min, sedimented by centrifugation at 20 000 x *g* for 1 min, rinsed three times with water, resuspended in 10 μl of water, and dried on a glass slide to be examined by a light microscope (Zeiss, SteREO Discovery V8) equipped with light polarizers.

### CR spectral shift

We followed protocols described by Klunk *et al*. [39] to determine which aggregates display a red shift in their spectra after binding to CR. A 7.5 μl sample of each peptide or buffer alone (25mM sodium phosphate pH 7.4, 0.1M NaCl) was added to 7.5 μl of 300 μM CR, 25 mM phosphate, pH 7.4, 0.1M NaCl. All specimens were incubated at 37°C for 30 min. Spectra were recorded immediately after incubation using a NanoDrop ND-100 Spectrophotometer (Thermo Scientific, Inc.). To account for contributions from light scatter, the spectra of untreated peptides were subtracted from the corresponding spectra collected from peptides treated with CR.

### Fourier Transform Infrared Spectroscopy (FT-IR)

For FT-IR, (GA)6, (GP)6 and (GR)6 were dissolved to 10 mM in 25 mM sodium phosphate pD 7.5, 0.1M NaCl prepared with deuterated water. Immediately after solubilizing, fresh (GA)6, (GP)6 and (GR)6 were frozen in liquid nitrogen. Aged samples dissolved in deuterated buffer were incubated at 37°C with agitation in an EchoTherm Orbital mixer (level 9, Torrey Pines Scientific) for 6–8 days, followed by flash freezing in liquid nitrogen. Both fresh and aged samples were lyophilized prior to FT-IR measurements. Spectra were collected at room temperature in a Perkin-Elmer FT-IR equipped with attenuated total reflectance (ATR). 128 scans were accumulated and averaged for each spectrum at a resolution of 4 cm^-1^. Spectra were corrected for absorption of buffer by subtracting the spectrum of buffer alone.

### Primary neuron culture, transfection, and imaging

Primary mixed cortical neurons were dissected from embryonic day 20 rat pups and cultured at 0.6 x 10^6^ cells/ml, as described previously [40, 41]. Four days after plating, neurons were transfected with pGW1-mApple [40, 41] using Lipofectamine 2000 (Invitrogen, 52887), and peptides (dissolved in 0.1M NaCl, 25 mM sodium phosphate pH 7.4), were applied at a final concentration of 1mM (diluted from 20mM for (GA)3, (GP)3 and (GR)3) or 0.5mM (diluted from 10mM for (GA)6, (GP)6 and (GR)6). “Fresh” peptides were applied to cells immediately after dissolving in solution whereas “aged” samples were applied after 6–8 days of agitation as described above. To avoid contamination, peptides were not subjected to sonication. Longitudinal imaging was initiated 24 hours post-transfection, and accomplished using an automated fluorescence microscopy platform as before [40, 41]. Briefly, neurons were imaged using a Nikon TiE-B inverted microscope equipped with a high-numerical aperture 20X objective lens, a PerfectFocus3 system, an ASI2000 stage with rotary encoders in the x- and y-axes, a Lambda XL Xenon lamp (Sutter), and an Andor iXon 897 electron-multiplied charge coupled device (EMCCD) digital camera. All stage, shutter, and filter wheel movements were coordinated by code written in publicly-available software (μManager and ImageJ). Image processing and survival analysis were accomplished by original code written in Python or the ImageJ macro language. All statistical analyses were performed in R using the survival analysis package, or Prism (GraphPad). Differences in survival between populations of neurons were determined using the log-rank test (for 2 conditions) or Cox proportional hazards analysis (for > 2 conditions).

### Animal work

All vertebrate animal work was approved by the Committee on the Use and Care of Animals at the University of Michigan. All experiments were carefully planned so that we use as few animals as possible. Pregnant female wild-type, non-transgenic Long Evans rats (*Rattus norvegicus*) were housed singly in chambers equipped with environmental enrichment. They were fed ad libitum a full diet (30% protein, 13% fat, 57% carbohydrate; full information available at www.labdiet.com), and cared for by the Unit for Laboratory Animal Medicine (ULAM) at the University of Michigan. Veterinary specialists and technicians in ULAM are trained and approved in the care and long-term maintenance of rodent colonies, in accordance with the NIH-supported Guide for the Care and Use of Laboratory Animals. All rats were kept in routine housing for as little time as possible prior to euthanasia and dissection, minimizing any pain and/or discomfort. Pregnant dams were euthanized by CO_2_ inhalation at gestation day 20. For each animal, euthanasia was confirmed by bilateral pneumothorax. Euthanasia was fully consistent with the recommendations of the Guidelines on Euthanasia of the American Veterinary Medical Association and the University of Michigan Methods of Euthanasia by Species Guidelines. Following euthanasia, the fetuses were removed in a sterile manner from the uterus and decapitated. Primary cells from these fetuses were dissected and cultured immediately afterwards, as described above.

### Fluorescent labeling of peptides

A stock solution of Fluorescein-5-EX succinimidyl ester (FITC, Molecular Probes, F6130) was prepared by dissolving the reagent in DMSO to a final concentration of 20mM. (GA)6, (GP)6 and (GR)6 were dissolved in 100mM sodium bicarbonate pH 8.5 to 1.5mg/ml. FITC was added to (GA)6, (GP)6 and (GR)6 solution to a final concentration of 8.3mM, 7.0mM and 5.3mM, respectively. All reaction volumes were adjusted to 800 μl. The final concentration the peptides was 0.75mg/ml. To make the unlabeled (control) solution, 400 μl of 100 mM sodium bicarbonate pH 8.5 was mixed with 400 μl of 20 mM FITC stock. All solutions were incubated for 24 hours at 4°C on a nutator. The labeled peptides were dialyzed against 2 L of 25 mM sodium phosphate pH 7.4; 0.1M NaCl. The dialysis buffer was changed four times. Peptide labeling was confirmed with MALDI-TOF (Bruker AutoFlex Speed). After 3 days of dialysis, samples were incubated for a week at 37°C with shaking in EchoTherm Orbital mixer (level 9, Torrey Pines Scientific).

### Detection of internalized FITC-labeled peptides

Following conjugation of each DPR to FITC, labeled DPRs were applied to primary rodent cortical neurons on DIV4 at a final concentration of 37.5 μg/ml. The cells were subjected to immunocytochemistry, as described below, using primary antibodies against MAP2 (dilution 1:500, Millipore, MAB3418, mouse monoclonal, clone AP20) and secondary anti-mouse Cy5-conjugated antibodies (dilution 1:250, Jackson Immunoresearch, 115-175-146, Goat whole IgG). The cells were then imaged using a Zeiss LSM510 laser scanning confocal microscope. For micrographs of neurons treated with FITC-labeled peptides, 20 optical slices were taken for each cell with a step size of 5 nm between slices. Z-sections were constructed using LSM Browser software (Zeiss). A total of > 200 neurons were counted from each population to determine the frequency of internalization.

### Generation and characterization of dipeptide repeat antibodies

Rabbit polyclonal antibodies were generated commercially by NeoScientific. Antisera were generated against synthetic (GA)6 or (GR)6 peptides and were affinity purified prior to use. Western blot validation was done on cell lysates from transfected COS-7 cells (purchased from ATCC) as previously described [42]. Briefly, COS-7 cells were lysed in RIPA (50mM Tris, pH8, 150mM NaCl, 0.1% SDS, 1% NP-40, 0.5% sodium deoxycholate) buffer supplemented with Complete mini protease inhibitor cocktail tablets (Sigma/Roche, 11836153001) and passed through a 27-gauge needle to shear DNA. Equal amounts of protein were run on a 12% SDS polyacrylamide gel. After transfer to PVDF membrane (0.2μm, Bio-Rad, 162–0177), blots were incubated with the following antibodies: mouse anti-V5 (Abcam, ab27671, mouse monoclonal, IgG2a; recognizes a Pk eptope in P/V proteins of the paramyxovirus SV5; dilution 1:1000), rabbit anti-GA (dilution 1:100) or rabbit anti-GR (dilution 1:5000) or mouse anti-β-Actin (Sigma, A1978, mouse monocolonal, IgG1; recognizes an epitope located in the N-terminus of the β-isoform of actin; dilution 1:5000). At least three independent experiments were performed and scanned films were processed and quantified using ImageJ software. Peroxidase-AffiniPure Goat Anti-Rabbit IgG (H+L) (Jackson ImmunoResearch, 111-035-144; polyclonal; conjugated to Horseradish Peroxidase) and Peroxidase AffiniPure Goat Anti-Mouse IgG (H+L) (Jackson ImmunoResearch, 115-035-146; polyclonal; conjugated to Horseradish Peroxidase) were used as secondary antibodies. For immunofluorescence based validation, COS-7 cells were maintained 37°C in 5% CO_2_ incubators. Dulbecco’s modified Eagle’s medium (DMEM, Fisher, SH30022FS) supplemented with 10% fetal bovine serum (Fisher, MT35015CV), and 1% Pen-Strep were used as culture media. Cells were transfected using Lipofectamine LTX with Plus Reagent (Thermo Fisher, 15338100) using manufacturer’s protocol. At 48 hours after transfection, cells were fixed with 4% paraformaldehyde for 15 minutes, washed, permeabilized with 0.1% triton X-100, blocked with 5% normal goat serum (NGS, Vector labs, S-1000) in 1X PBS containing 0.1% triton X-100, and incubated with mouse anti-GFP (Roche, 11814460001, mixture of clones 7.1 and 13.2 mouse monoclonal antibodies, IgG1k, which recognizes wild type and mutant forms of GFP; dilution 1:1000), rabbit anti-GA (dilution 1:100) or anti-GR (dilution 1:500) overnight. Slides were washed and probed with Goat anti-Mouse IgG (H+L) Secondary Antibody, Alexa Fluor 488 (Thermo Fisher Scientific, A-11001, goat polyclonal, immunogen: gamma immunoglobins heavy and light chains) and Alexa Fluor 555 labeled Goat-anti-Rabbit (Thermo Fisher Scientific, A-21428, goat polyclonal, immunogen: gamma immunoglobins heavy and light chains) antibodies and visualized with an inverted Olympus IX71 epifluorescence microscope with Slidebook software with identical fluorescent settings for each slide

Immunohistochemical validation of the antibodies was done as in Todd *et al* [42]. Briefly, human cerebellum from control cases or cases with *C9orf72* expansions confirmed by repeat primed PCR testing were deparaffinized and then processed through a basic antigen retrieval protocol to enhance detection. Sections on slides were then permeabilized with 0.1% triton X-100, blocked in 5% NGS and 0.1% BSA, and incubated in primary antibodies overnight at 4°C at the following concentrations: anti-GA (1:50), anti-GR (1:100). Following PBS washes, a Vectastain Elite ABC Kit (Rabbit IgG) (Vector Labs, PK-6101) was used according to manufacturer’s protocols for DAB staining. Samples were counterstained with hematoxylin QS (Vector Labs, H-3404) to identify nuclei. Selected images are representative of staining from a least three different slides and two patients per group.

### Detection of peptide internalization by immunocytochemistry

We added 0.5 mM (final concentration) of fresh or aged (GA)6, (GP)6 and (GR)6 to primary neurons (cultured as described above) 4 days after dissection (DIV4). After 24 hours, the cells were rinsed twice in PBS (Life Technologies, 70011–044), then fixed in 4% paraformaldehyde for 10 min. Following 2 more rinses, neurons were permeabilized with 0.1% Triton X-100 in PBS for 20 min at room temperature, equilibrated with 10 mM glycine in PBS for 10 min at room temperature, then blocked in 0.1% Triton X-100, 3% BSA (Research Products International, 9048-46-8) and 0.2% goat serum in PBS for 1 hour at room temperature. Primary antibodies against GA (1:100) and GR (1:500), both generated as described above, or GP (1:5000, polyclonal, Millipore ABN455, rabbit polyclonal), in addition to antibodies against MAP2 (1:500, Millipore MAB3418, mouse monoclonal, clone AP20), were added directly to the block and the samples incubated overnight at 4°C. All cells were rinsed twice quickly and 3 times for 10 min with PBS, then placed back in block solution containing the appropriate secondary antibodies (goat anti-mouse Cy5, Jackson ImmunoResearch, 115-175-146, whole IgG; and goat anti-rabbit Alexa Fluor 488, Life Technologies A-11008, whole IgG) at a dilution of 1:250. The cells were rinsed twice quickly in PBS, and 3 times for 10 min each in PBS containing Hoescht dye (Invitrogen, 33342,) at 100 nM, then twice more in PBS before imaging by automated microscopy. A total of > 100 neurons were counted from each population to determine the frequency of internalization.

## Results

In humans carrying pathogenic *C9orf72* mutations, several RAN translation products differing in primary sequence and length may be produced simultaneously [16]. To assess the properties of different DPRs, we studied individual peptides with either three or six repeats of GA, GP or GR motifs. These three motifs correspond to the expected sense translation products of the mutant (GGGGCC)_n_ repeat sequence.

Longer peptides, in particular GA-containing peptides were difficult to synthesize and displayed a high tendency to precipitate from solution. To assess how aging affects the properties of the aggregates, we analyzed peptides immediately after solubilizing (fresh) or after incubation at 37°C for a week (aged). When dissolving peptides, we opted for a buffer that would be innocuous to neurons (phosphate-buffered saline) and sonicated the peptides to facilitate their solubilization rather than using organic solvents.

### Glycine-proline peptides are resistant to aggregation and nontoxic

**Fig 1:**
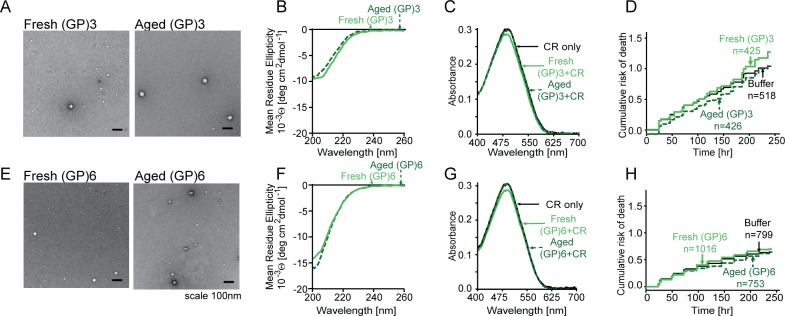
GP dipeptides form amorphous aggregates that are innocuous to neurons. (A,E) Fresh and aged (GP)3 and (GP)6 peptides formed low abundance amorphous aggregates by TEM. (B,F) CD spectra of fresh and aged (GP)3 and (GP)6 were indistinguishable from one another. (C,G) Neither (GP)3 nor (GP)6 peptides exhibited affinity for CR, and their absorbance spectra did not change in the presence of CR. (D,H) Cumulative hazard plots for primary cortical neurons exposed to (GP)3 and (GP)6 peptides. Neither (GP)3 nor (GP)6 significantly impacted neuronal survival (p > 0.05) compared to buffer alone. n, number of neurons per condition. Survival data were pooled from 8 wells per condition, in each of 3 replicates.

By TEM, we observed only sparse, round shaped aggregates in fresh and aged samples of peptides with 3 or 6 repeating GP motifs (Fig 1A and 1E). The CD spectra of fresh and aged (GP)3 and (GP)6 were indistinguishable, resembling the spectra of other poly-proline peptides (Fig 1B and 1F, Table 1) [43]. The FT-IR spectra of soluble and aged (GP)6 were also similar (S1 Fig). Additionally, the absorbance spectrum of CR, a marker of amyloid aggregates, did not change in the presence of (GP)3 or (GP)6 (Fig 1C and 1G). Thus, under the conditions tested GP peptides formed only sparse aggregates and we did not observe any difference between fresh and aged GP- containing samples.

**Table 1:** Secondary structure content of the peptides in this study calculated from their corresponding CD spectra [38].

Peptide		Helix [%]	Sheet [%]	Turn [%]	Unordered [%]	Range of data used for the predictions [nm]*
(GA)3	Fresh	4	44	21	31	195–260
	Aged	4	44	21	31	195–260
(GP)3	Fresh	7	32	24	37	190–260
	Aged	5	30	25	40	190–260
(GR)3	Fresh	4	41	21	34	200–260
	Aged	4	41	21	34	200–260
(GA)6	Fresh	2	50	19	28	190–260
	Aged	1	55	18	27	190–260
(GP)6	Fresh	7	29	25	40	190–260
	Aged	6	23	27	44	190–260
(GR)6	Fresh	4	40	21	35	200–260
	Aged	4	40	21	35	200–260

*The range specifies the wavelength range of the data which was used for the predictions.

GP-containing peptides showed little toxicity in longitudinal assessments of neuronal survival. For these assays, we applied fresh or aged (GP)3 and (GP)6 peptides to rodent primary cortical neurons and tracked the survival of neurons using a fully-automated microscopy platform [40, 41]. Neurons were transfected with a vector encoding mApple and imaged sequentially at 24-hour intervals. Individual cells were identified and tracked by custom-written image analysis algorithms and time of death was determined by changes in neuronal morphology or loss of fluorescence, sensitive measures of cell death in previous studies [40, 41, 44]. Using Cox proportional hazards analysis, these data were used to generate cumulative hazard plots and hazard ratios (HRs), representing relative risks of death in comparison to a reference population (cells treated with buffer alone). Neither fresh nor aged GP samples were toxic when applied to primary neurons (HR 0.98–1.14, p>0.05; Fig 1D and 1H). Thus, (GP)3 and (GP)6 rarely aggregate and are nontoxic in this model system, in accord with prior results [17, 19, 24].

### Glycine-arginine dipeptides form spherical neurotoxic aggregates

**Fig 2:**
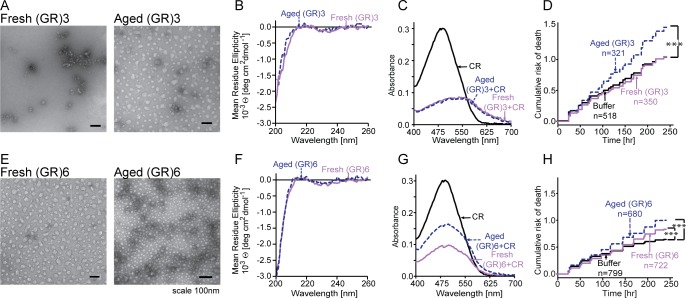
GR dipeptides form repeat length-independent neurotoxic aggregates. **(A,E)** The majority of aged (GR)3 and (GR)6 aggregates were spherical in shape, as assessed by TEM. **(B,F)** CD spectra of fresh and aged (GR)3, as well as fresh and aged (GR)6, were indistinguishable. **(C,G)** (GR)3 peptides produced a spectral shift from 490nm to 550nm and decreased the maximum of CR absorbance at 490nm. CR spectra in the presence of fresh and aged (GR)3 were indistinguishable. In contrast, absorbance spectra of fresh and aged (GR)6 differed, with fresh peptide showing greater decrease in absorbance. **(D,H)** In longitudinal analyses of neuronal survival, aged but not fresh (GR)3 significantly increased the risk of death over control neurons exposed to buffer alone, whereas both fresh and aged (GR)6 were significantly toxic. n, number of neurons per condition. *** p< 0.0003, Cox proportional hazards analysis. Survival data were pooled from 8 wells per condition, in each of 3 replicates.

Arginine-containing dipeptides demonstrated toxicity in several cell culture and animal models of C9ALS/FTD [19, 21, 23–26, 29]. Fresh and aged (GR)3 peptides formed spherical aggregates by TEM, and aged (GR)3 samples appeared more homogeneous than the fresh samples (Fig 2A). The CD spectra of fresh and aged (GR)3 were similar and contained a small negative band centered at 230 nm that is often associated with type I β-turns (Fig 2B and 2F) [45]. Secondary structure predictions indicated that about 55% of the protein exhibits turn and/or random coil conformation and 41% is β-sheet (Table 1). Both fresh and aged peptides equally reduced CR absorbance and there was no apparent difference in their CR spectra (Fig 2C). However, aged but not fresh (GR)3 significantly increased the risk of death in primary rodent cortical neurons (HR 1.36, p = 3.5x10^-4^) (Fig 2D). The inability of (GP)3 and (GP)6 to elicit toxicity in similar assays (Fig 1D and 1H) argues against a nonspecific reduction of neuronal survival caused by the quantities of (GR)3 used in these studies.

Similar to aged (GR)3, fresh and aged (GR)6 samples displayed spherical morphology by TEM (Fig 2E). CD spectra for (GR)6 demonstrated that, like (GR)3, a large proportion of the peptide (56%) is turn or random coil and approximately 40% of fresh and aged (GR)6 samples were predicted to be in a β-sheet conformation (Fig 2F and Table 1). As revealed by TEM, a large portion of the (GR)6 sample is present within aggregates, which often reduces the measured CD signal. Accordingly, we utilized FT-IR, which has previously been used for secondary structure determination of protein aggregates [46]. With FT-IR, fresh and aged samples were nearly identical to one another, suggesting that there are no changes in their secondary structure (S1 Fig). (GR)6 also bound CR, with aged samples reducing the absorption maximum of CR to a lesser extent than fresh ones (Fig 2G). Both fresh and aged (GR)6 increased the risk of death for rodent primary cortical neurons in longitudinal assays (HR 1.2, p = 4.8x10^-3^, and HR 1.5, p = 6.6x10^-9^, respectively) (Fig 2H). In comparison to fresh (GR)6, aged peptide was significantly more toxic (HR 1.25, p 0.001). Thus, aged GR dipeptides, regardless of their length, formed aggregates with spherical morphology that elicited toxicity when applied to neurons.

### (GA)6 forms atypical amyloid-like structures and is toxic to neurons

**Fig 3:**
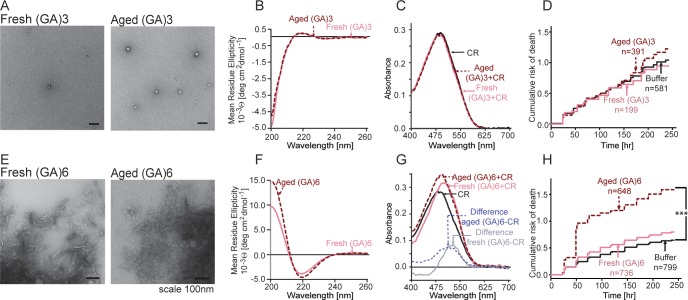
GA dipeptides form repeat length-dependent neurotoxic β-sheet rich aggregates. **(A,E)** By TEM, (GA)3 formed sparse amorphous aggregates. Fresh (GA)6 aggregates were heterogeneous, containing both fibrils and layered sheets. In contrast, aged (GA)6 formed large aggregates mostly composed of layered sheets. **(B,F)** CD spectra of fresh and aged (GA)3 were similar with 44% predicted β-sheet content. Aged (GA)6 had the largest β-sheet content among the peptides in this study (55%). **(C,G)** No change in the CR absorbance spectrum was noted for (GA)3, but fresh and aged (GA)6 displayed increased CR absorbance, as is commonly observed for amyloid-forming proteins. Fresh and aged samples introduced spectral shifts to 530nm and 515 nm, respectively. **(D,H)** Aged (GA)6 peptides, but not (GA)3 peptides, were highly toxic to neurons compared to buffer only control. n, number of neurons per condition. *** p < 0.0003, Cox proportional hazard analysis. Survival data were pooled from 8 wells per condition, in each of 3 replicates.

Fresh and aged (GA)3 were indistinguishable by TEM, both forming rare amorphous aggregates (Fig 3A). CD spectra of fresh and aged (GA)3 were also similar (Fig 3B). Neither fresh nor aged (GA)3 demonstrated detectable binding to CR (Fig 3C), and neither sample reduced neuronal survival in longitudinal studies (HR 0.86–1.09, p>0.05; Fig 3D).

Increasing the number of GA repeats from 3 to 6 had a striking effect on the structure of the peptide. By TEM, aged (GA)6 samples displayed large aggregates consisting of multilayered flat sheets (Fig 3E), whereas fresh (GA)6 samples contained fibrillar aggregates in addition to the flat sheets. Moreover, secondary structure predictions indicated a 5% increase in β-sheet content for aged (GA)6 compared to fresh (GA)6 (Fig 3F and (Table 1) [38]. Similarly, FT-IR spectra contained bands at 1622cm^-1^ and 1698cm^-1^, reminiscent of anti-parallel β-sheet (S1 Fig) [47]. As with CD spectra, we noticed variations in FT-IR spectra of fresh versus aged (GA)6.

Both fresh and aged (GA)6 introduced a shift in the CR spectrum and an increase in overall CR absorbance, changes that are common for amyloid aggregates [39]. This shift in CR absorbance is highlighted by the residual absorbance of the difference spectra, calculated by subtracting the baseline CR spectra from the CR spectra collected in the presence of (GA)6 (Fig 3G). Intriguingly, we observed a difference between the maximum residual absorbance produced by fresh (GA)6 (530 nm) and aged (GA)6 (515 nm). The shift demonstrated by fresh (GA)6 is similar to that produced by amyloid-β fibrils [39].

While both fresh and aged (GA)6 samples significantly reduced neuronal survival, the magnitude of the effect was much greater for aged (HR 2.88, p = 2x10^-16^) than for fresh (GA)6 samples (HR 1.29, p = 3.3x10^-4^; Fig 3H). Thus, GA dipeptides displayed a strong correlation between β-sheet content and neuronal toxicity.

**Fig 4:**
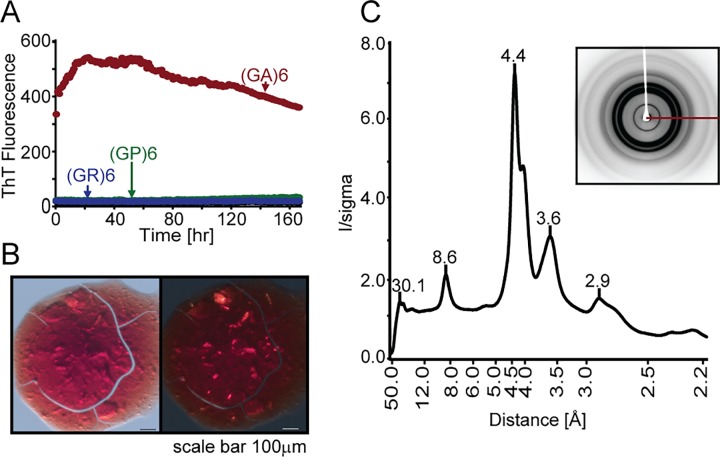
(GA)6 forms atypical amyloid-like aggregates. (A) ThT binding assays for all peptides, demonstrating significant binding only for (GA)6 (red line). (B) Aged (GA)6 in the presence of CR appeared red with unpolarized light (left) and displayed yellow birefringence with cross-polarized light (right). (C) X-ray fiber diffraction pattern from (GA)6 peptides suggests that the aggregates assume a discrete structure.

To further explore whether DPRs share features with amyloids, we assessed ThT binding for each of the peptides studied here. None of the shorter DPRs displayed detectable ThT binding (data not shown). Only the most toxic peptide, (GA)6, exhibited affinity for ThT (Fig 4A), which is a common feature of amyloid aggregates. In addition, aged (GA)6 displayed yellow birefringence in the presence of CR (Fig 4B), consistent with other amyloid-like aggregates [48].

Amyloid aggregates also exhibit a distinct x-ray diffraction pattern elicited by the unique β-sheet conformation typical of amyloid [49]. As shown in Fig 4C, the x-ray fiber diffraction pattern of aged (GA)6 contains a strong, sharp 4.4 Å reflection and a broader, weaker 8.6 Å reflection. However, the strand-to-strand spacing implied by the 4.4 Å reflection of (GA)6 is smaller than the 4.8 Å typical of β-sheet structures formed by amyloids. Furthermore, the 8.6 Å reflection is different than the 3.9 Å - 5.3 Å spacing expected for β-sheets composed of glycine and alanine (Fig 4C). Thus, the diffraction pattern for (GA)6 suggests a highly-ordered structure.

### Externally applied arginine- and alanine-containing peptides are internalized by neurons

RAN translation products are usually detected within cytoplasmic inclusions of C9ALS/FTD brain tissue and are not typically observed extracellularly [16]. However, some amyloidogenic proteins, such as amyloid β_1–42_, bind lipid membranes and extracellular receptors to trigger toxic signal transduction cascades [50]. Recent evidence also suggests that some aggregation-prone peptides and proteins may pass from one neuron to the next through endocytic and exosomal pathways [51, 52].

We therefore sought to determine whether externally applied DPRs could be internalized by neurons in culture. For detection of GP, we used commercially available antibodies, but for GA and GR peptides we developed specific anti-DPR antibodies. Polyclonal rabbit antibodies were raised against synthetically-generated (GA)6 or (GR)6 peptide antigens. These antibodies were validated for specificity first by Western blot and ICC in cells transfected with ATG-V5- GA-GFP and ATG-V5- GR-GFP fusion proteins (S2 Fig). Both antibodies also recognized V5-reactive specific bands at different repeat sizes. Moreover, ICC using anti-DPR and -GFP antibodies showed significant co-localization of both antibodies with the epitope tag. To determine if these antibodies are capable of recognizing DPR aggregates in their native state, we performed IHC in C9ALS/FTD brain tissues and controls. Both GA and GR stained perinuclear aggregates in patient brains but not in controls (S2 Fig), consistent with published results [8, 9].

**Fig 5:**
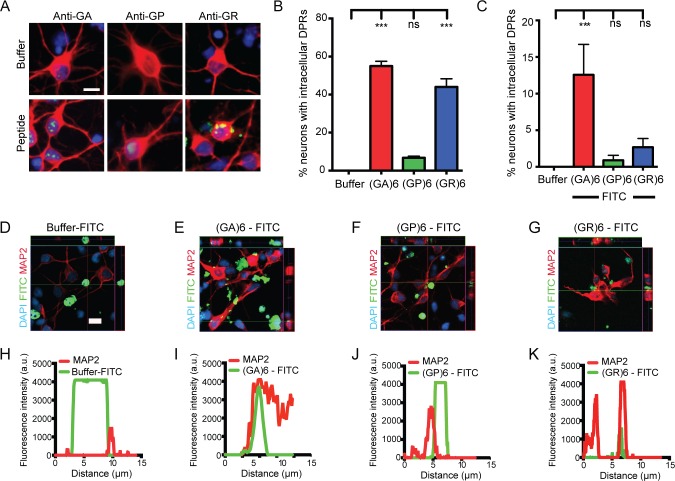
DPRs are internalized by neurons. (A) Fluorescence micrographs of neurons treated with the indicated peptide and stained with antibodies recognizing the pan-neuronal marker (MAP2, red), the indicated DPR (GA, GP or GR; green), and a nuclear dye (4’,6-diamidino-2-phenylindole (DAPI), blue). Scale bar, 20 **μ**m. (B) Frequency of DPR internalization detected by immunocytochemistry. (C) Quantification of the data in panels D-G, showing internalization of FITC-labeled DPRs. (D-G) Confocal microscopy of neurons treated with the indicated peptide shows intracellular accumulations of FITC-(GA)6 and -(GR)6, but not -(GP)6 or buffer. Representations of the y-z axis appear above, and x-z plots to the right of each micrograph. Scale bar in (D), 20 **μ**m, applies to (D-G). (H-K) Intensity plots of fluorescence across a line that intercepts the inclusions shown in D-G, demonstrating overlapping MAP2 and FITC signals for (GA)6 and (GR)6 but not (GP)6. For (B) and (C), ***, p<0.05 one-way ANOVA with Dunnett’s test. n = 100–150 neurons per condition, pooled from 3 replicates and 2 independent experiments. ***, p<0.05 one-way ANOVA with Dunnett’s test.

To determine if applied DPRs were internalized, primary neurons in culture were fixed 24h after peptide application, immunostained with antibodies recognizing each of the DPRs (GA, GP and GR) and imaged by automated microscopy (Fig 5A; S3 Fig). Both nuclear and cytoplasmic inclusions were detected in several neurons treated with aged (GA)6 and (GR)6. In contrast, diffuse nuclear staining was noted in a small proportion of cells exposed to (GP)6, but the vast majority had no evidence of staining over background. Furthermore, DPR internalization was significantly more frequent for (GA)6 and (GR)6 than for (GP)6 (Fig 5B), in accord with the toxicity of (GA)6 and (GR)6 in neurons. We did not detect intracellular DPRs in neurons treated with buffer alone.

To confirm these results, we also covalently coupled a fluorescent molecule, fluorescein-5-EX, succinimidyl ester (FITC), to aged (GA)6, (GP)6 and (GR)6. After applying the aged, labeled peptides to primary neurons in culture, the cells were fixed, immunostained with an antibody recognizing the pan-neuronal cytoplasmic marker MAP2, and imaged by laser scanning confocal microscopy (Fig 5D–5G). Intracellular inclusions were detected in neurons treated with FITC-(GA)6 and FITC-(GR)6, but not FITC-(GP)6 or in cells treated with FITC alone, in agreement with the lack of toxicity of GP dipeptides (Fig 1H). Linear quantifications of the Z-sections shown in Fig 5D–5G demonstrated that the FITC-(GA)6 and FITC-(GR)6 peptides were indeed cytoplasmic, and not simply located along the extracellular face of the plasma membrane (Fig 5E–5H). In addition, intracellular deposits of FITC-(GA)6 were significantly more frequent than deposits of FITC-(GP)6 or FITC-(GR)6 (Fig 5C), consistent with the results obtained using anti-DPR antibodies (Fig 5B). DPR internalization occurred less frequently with FITC-labeled peptides than with unlabeled DPRs, and the distribution of internalized FITC-labeled peptides was also different, perhaps due to the size or charge of the FITC moiety. Nevertheless, these data suggest that (GA)6 and (GR)6 peptides are efficiently taken up by neurons, and that DPR internalization correlates with toxicity.

## Discussion

Here we studied the structural characteristics of the three sense-strand derived DPRs that accumulate and aggregate in C9ALS/FTD. We demonstrate that different DPR peptides exhibit marked differences in their structural features and in their propensity to form aggregates. We also observed a correlation between these structures, their ability to enter cells, and their toxicity when applied exogenously to neurons. These findings suggest that specific structural conformations formed by GA- and GR- repeat proteins may be elemental to their toxicity and could serve as a target for future therapeutic strategies.

Our findings suggest that the length of the dipeptide repeat can influence aggregate structure, internalization and downstream neurotoxicity in a simple model system. Although RAN peptides produced *in vivo* from *C9orf72* expansions may be significantly longer than those studied here, our data show that even short DPRs consisting of 3–6 repeat motifs formed amyloid-like structures and impaired neuronal survival, suggesting that these elements are sufficient for aggregation and neurotoxicity. Consistent with our results, Chang *et al*. [14] showed that externally-applied GA-containing peptides with fifteen repeats display features of amyloid, are taken up by cells, and are toxic to transformed cell lines. Thus, the ability of relatively short peptides to cause toxicity suggests that these peptides could accelerate neurodegeneration if present at high local concentrations.

In individuals with *C9orf72* mutations, RAN proteins are likely produced in multiple reading frames within the same cell, thus the relevance of the exogenously applied individual dipeptide toxicity observed here to that in the human disease remains to be confirmed. However, RAN proteins are detectable in the cerebrospinal fluid of patients with *C9orf72* mutations [53]. Given emerging evidence for prion-like spread of aggregation-prone proteins in other neurodegenerative disorders, these studies suggest that DPRs could enhance disease progression by catalyzing the spread of toxicity from one cell to another [14, 51, 52, 54].

Of the peptides studied, (GA)6 and (GR)6 formed β-sheet structures, were taken up most effectively by neurons, and demonstrated toxicity in longitudinal assays, suggesting a potential relationship between structure, internalization, and cell death (Fig 5). In contrast, GP-containing peptides formed amorphous aggregates, were not internalized by neurons, and were not toxic despite the relatively high concentrations used here. In prior investigations, intrinsically overexpressed GA DPRs form cytoplasmic aggregates, while overexpressed GR DPRs accumulate within nuclear foci [17, 21, 23, 24, 26]. In contrast, we detected externally applied, synthetic GR DPRs within the cytoplasm of neurons, and GA DPRs within the nucleus. The apparent discrepancy may be due to the method of delivery, since other investigations noted a similar cytoplasmic deposition of arginine-rich DPRs when applied externally to cells in culture [28]. While no previous study examined the subcellular localization following internalization of synthetic GA DPRs, the nuclear localization of (GA)6 resembles the nuclear aggregation of other proteins containing polyalanine stretches [55].

Arginine-containing RAN translation products elicit toxicity in cellular and animal models of disease when exogenously applied or when intrinsically expressed under the control of a high-level promoter preceded by an AUG start codon [17, 19, 21–26, 53]. Our results extend these observations, demonstrating that peptides containing three (GR) repeats form aggregates with distinct spherical morphology and cause neuronal death. Although prolonged incubation enhanced the toxicity of GR- containing peptides, the secondary structure and the morphology of the aggregates remained unchanged. However, we observed that fresh and aged (GR)6 have different affinities for CR, possibly reflecting subtle structural changes within the aggregates. Higher resolution studies will be required to reveal the packing of peptides within the aggregates that could account for the acquired toxicity.

Using several independent measures, we found that aged (GA)6 shares key properties with amyloid while remaining structurally distinct. Diffraction from amyloid fibrils typically yields a cross-β pattern, featuring two strong reflections at resolutions corresponding to strand-to-strand and sheet-to-sheet distances. If aged (GA)6 forms amyloid, we would predict a small sheet-to-sheet spacing consistent with the size of the glycine and alanine side chains (3.9 Å, and 5.3 Å, respectively). However, we did not observe either of these, nor did we observe a reflection at 4.8 Å, corresponding to the spacing between strands. Instead, the diffraction pattern shows strong reflections at 4.4, 3.6, and 8.6 Å (Fig 4C). One possible explanation is that (GA)6 assumes a cross-β diffraction pattern, but the reflection corresponding to strand-to-strand spacing is extinguished by the presence of a two-fold screw symmetry axis coincident with the fibril axis, as is common in crystals of amyloidogenic peptides with class 1 symmetry [36]. In this case, the 4.4 Å spacing would correspond to a set of Bragg planes, closely related to the strand-to-strand spacing but slightly offset in angle and spaced closer together, while the 8.6 Å spacing might arise from the distance between adjacent pairs of β-sheets. We also note that aged (GA)6 grows in large flat sheets, a morphology different from fibrillar aggregates formed by amyloids.

## Conclusions

In summary, our work defines unique structural elements for individual DPRs and correlates the abundance of specific structures with their cellular internalization and relative toxicity. The most neurotoxic dipeptide in our study, (GA)6, exhibits a distinct structure that is β-sheet rich and shares features with amyloid. This link between structure and neurotoxicity suggests that strategies targeting such conformations may effectively reduce RAN peptide-induced neurodegeneration and slow disease progression.

## Supporting Information

S1 Fig FT-IR spectra of dipeptide repeats. To assess for secondary structure, fresh and aged (GA)6, (GP)6 and (GR)6 peptides were subject to FT-IR. The two bands at 1622cm^-1^ and 1698cm^-1^ in (GA)6 spectra suggest that the secondary structure of these aggregates is mostly β-sheet.
(TIF)

S2 Fig Characterization of new C9orf72 dipeptide repeat antibodies. Rabbit polyclonal antibodies were generated against the sense strand-derived DPRs GA and GR. (**A**) Western blot of lysates from COS-7 cells transfected with the indicated vectors. Blots were serially probed with the indicated DPR antibody, then V5 and then actin as a loading control. (**B,C**) Immunocytochemistry of COS-7 cells transfected with the indicated vectors and stained for GR (**B**) and GA (**C**) antibodies demonstrate specificity. (**D**) Immunohistochemistry of GA- and GR- protein aggregates in the cerebellum of a genetically confirmed *C9orf72(+)* ALS case. No significant staining was observed in control patient cerebellum.
(TIF)

S3 Fig Detection of internalized DPRs by immunocytochemistry. 24 h after application of (GA)6, (GP)6 or (GR)6 peptides to live rodent primary cortical neurons, the cells were fixed and immunostained using antibodies against MAP2 (red) and each of the DPRs (green), and nuclei labeled with DAPI (blue). (**A**) Neurons treated with (GA)6 displayed nuclear foci. (**B**) Diffuse nuclear staining was noted in a small proportion of cells exposed to (GP)6. (**C**) Following application of (GR)6, DPRs were detected within neuronal cytoplasmic aggregates. See Fig 5B for quantification of DPR internalization. Scale bar, 20 μm.
(TIF)
