# Supplementary figures and images for: Distinct C9orf72-Associated Dipeptide Repeat Structures Correlate with Neuronal Toxicity
Source: PLoS One. 2016 Oct 24;11(10):e0165084. doi: 10.1371/journal.pone.0165084 (PMC5077081; doi:10.1371/journal.pone.0165084)

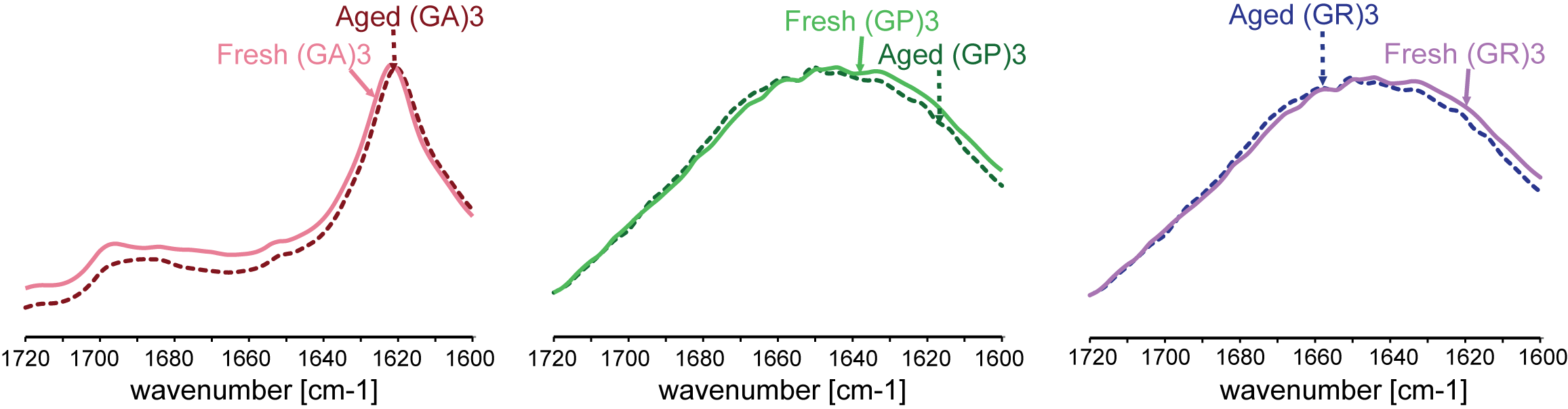

Supplement: S1 Fig — To assess for secondary structure, fresh and aged (GA)6, (GP)6 and (GR)6 peptides were subject to FT-IR. The two bands at 1622cm-1 and 1698cm-1 in (GA)6 spectra suggest that the secondary structure of these aggregates is mostly β-sheet. (TIF) [file pone.0165084.s001.tif]

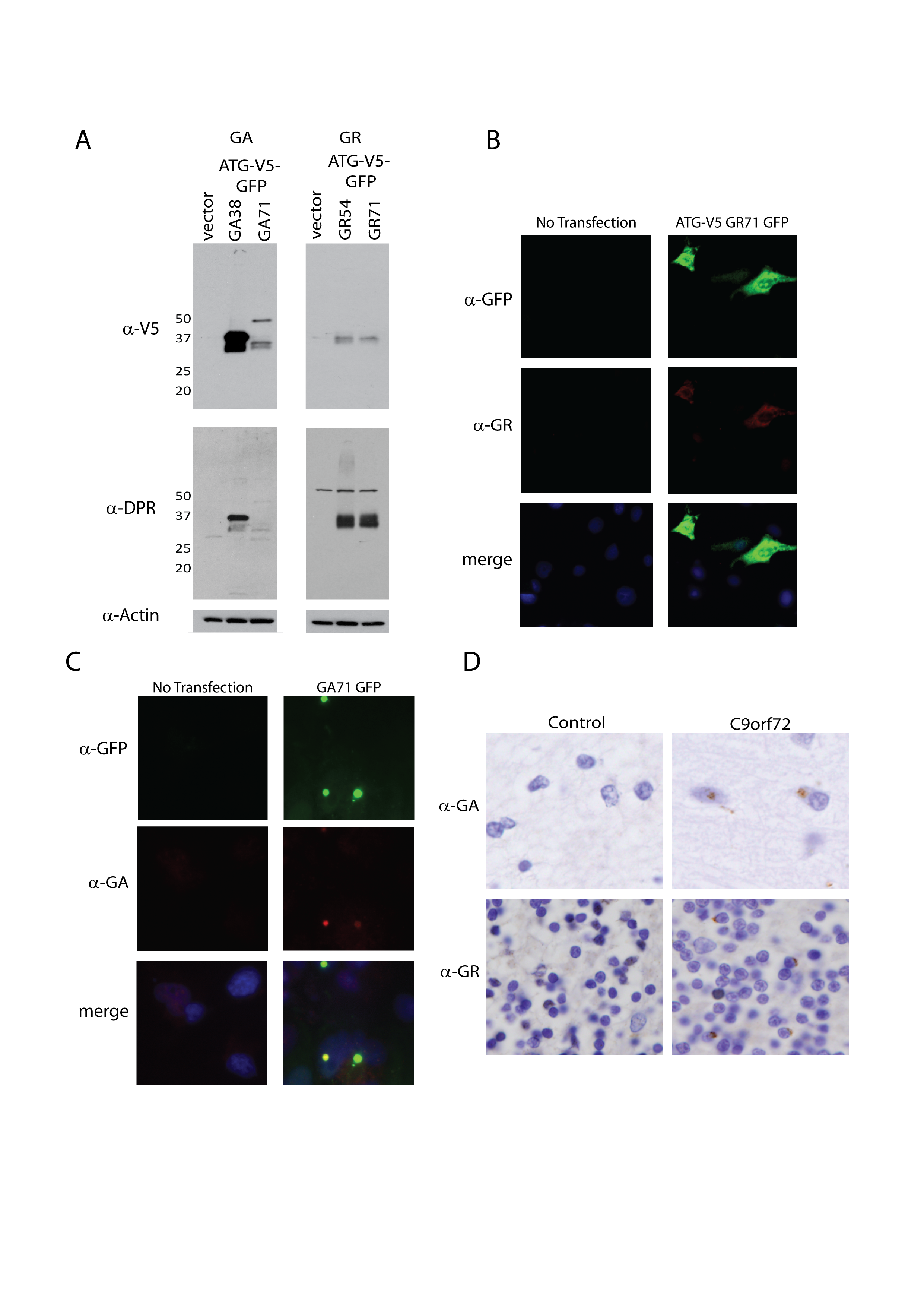

Supplement: S2 Fig — Rabbit polyclonal antibodies were generated against the sense strand-derived DPRs GA and GR. (A) Western blot of lysates from COS-7 cells transfected with the indicated vectors. Blots were serially probed with the indicated DPR antibody, then V5 and then actin as a loading control. (B,C) Immunocytochemistry of COS-7 cells transfected with the indicated vectors and stained for GR (B) and GA (C) antibodies demonstrate specificity. (D) Immunohistochemistry of GA- and GR- protein aggregates in the cerebellum of a genetically confirmed C9orf72(+) ALS case. No significant staining was observed in control patient cerebellum. (TIF) [file pone.0165084.s002.tif]

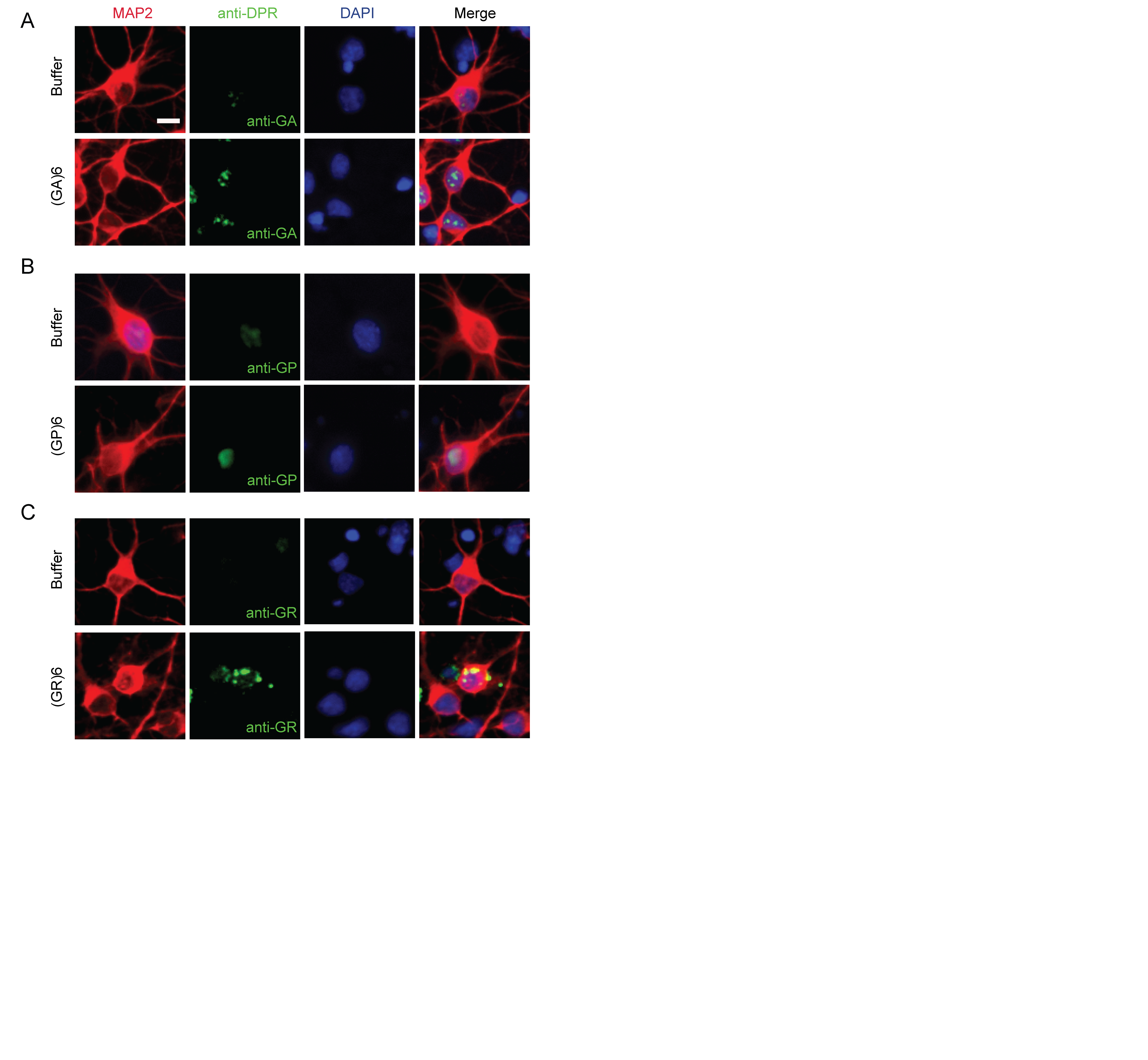

Supplement: S3 Fig — 24 h after application of (GA)6, (GP)6 or (GR)6 peptides to live rodent primary cortical neurons, the cells were fixed and immunostained using antibodies against MAP2 (red) and each of the DPRs (green), and nuclei labeled with DAPI (blue). (A) Neurons treated with (GA)6 displayed nuclear foci. (B) Diffuse nuclear staining was noted in a small proportion of cells exposed to (GP)6. (C) Following application of (GR)6, DPRs were detected within neuronal cytoplasmic aggregates. See Fig 5B for quantification of DPR internalization. Scale bar, 20 μm. (TIF) [file pone.0165084.s003.tif]
